# BTK inhibition in primary central nervous system lymphoma: mechanisms, clinical efficacy, and future perspectives

**DOI:** 10.3389/fonc.2024.1463505

**Published:** 2024-12-24

**Authors:** Yurou Xing, Kejia Zhao, Yi Zhang, Yongsheng Wang

**Affiliations:** ^1^ Thoracic Oncology Ward, Cancer Center, West China Hospital, Sichuan University, Chengdu, Sichuan, China; ^2^ Department of Thoracic Surgery and Institute of Thoracic Oncology, West China Hospital of Sichuan University, Sichuan University, Chengdu, Sichuan, China

**Keywords:** B-cell receptor signaling, Bruton’s tyrosine kinase (BTK) inhibitors, primary central nervous system lymphoma (PCNSL), mechanism, lymphoma

## Abstract

The prognosis of primary central nervous system lymphoma (PCNSL) patients is relatively poor, and there is currently no standard treatment plan. Most patients choose high-dose chemotherapy based on methotrexate. While traditional chemotherapy combined with biological therapy has achieved limited results, some patients still do not respond to treatment or cannot tolerate its toxicity and side effects. Bruton’s tyrosine kinase (BTK) is a key enzyme in B-cell receptor signaling, and its activation is critical for B-cell survival and proliferation. In recent years, BTK inhibitors have shown great potential in treating lymphomas derived from various B cells because of their strong targeting ability and relatively few side effects. They may also be a reasonable treatment choice for PCNSL. This article reviews the mechanism of action, clinical research, adverse reactions, and other issues of BTK inhibitors in treating PCNSL to provide a reference for individualized treatment of patients with PCNSL.

## 1 Introduction

Primary central nervous system lymphoma (PCNSL) accounts for 4% of primary central nervous system (CNS) tumors and 4% to 6% of extranodal lymphomas (1) and has a relatively worse prognosis than other extranodal diffuse large B-cell lymphomas. High-dose methotrexate (HD-MTX) has improved blood−brain barrier permeability, and immunochemotherapy regimens based on HD-MTX are currently the first-line treatment for PCNSL patients. However, recurrence still occurs in more than 50% of patients. The prognosis of relapsed/refractory (R/R) PCNSL remains extremely poor, and there is currently no unified salvage treatment plan. Diffuse large B-cell lymphoma can be divided into two subtypes: the germinal center B-cell-like (GCB) subtype and the activated B-cell-like (ABC) subtype. Most PCNSLs are activated B-cell-like (ABC) cells. This subtype selectively acquires mutations that target the B-cell receptor (BCR) and promote BCR signaling, which supports the development of BTK inhibitors in PCNSL (2).

The pathophysiological process of PCNSL involves numerous distinct genetic mutations, the most important of which are functional mutations in the genes encoding the CD79b molecule (*CD79B*), a subunit of the B-cell receptor (BCR), and the MYD88 innate immune signal transduction adaptor (*MYD88*), which is involved in Toll-like receptor (TLR) and interleukin receptor signaling (3). In normal B cells, the TLR and BCR signaling pathways cooperate to activate the nuclear factor kappa-light-chain-enhancer of activated B cells (NF-κB). Mutated MYD88 and CD79B activate the NF-κB signaling pathway, promoting B-cell survival and proliferation (4). The frequency of MYD88 mutations in PCNSL patients is 76% (5). This high frequency underscores the pivotal role of MYD88 in the pathogenesis and progression of PCNSL, influencing therapeutic strategies targeting the B-cell receptor signaling pathway. In contrast, mutations leading to the activation of the NF-κB signaling pathway are present in more than 90% of patients with PCNSL (6). These findings show that the activation of the NF-κB signaling pathway is the key driver of the occurrence of PCNSL, providing an important theoretical reference for the use of Bruton’s tyrosine kinase (BTK) inhibitors to treat PCNSL.

PCNSL also have the same immune evasion mechanism: gene inactivation of major histocompatibility complex (MHC) classes I and II and β2-microglobulin (B2M), leading to the loss of their synthesized proteins (7). By systematically reviewing the preclinical and clinical data reported for BTK inhibitors, this review focuses on the mechanism of action of BTK inhibitors in PCNSL and the results of existing clinical trials and discusses their therapeutic efficacy, challenges, and future development directions, aiming to provide new perspectives and broader possibilities for treating PCNSL.

## 2 BTK and BTK inhibitors

### 2.1 BTK

**Figure 1:**
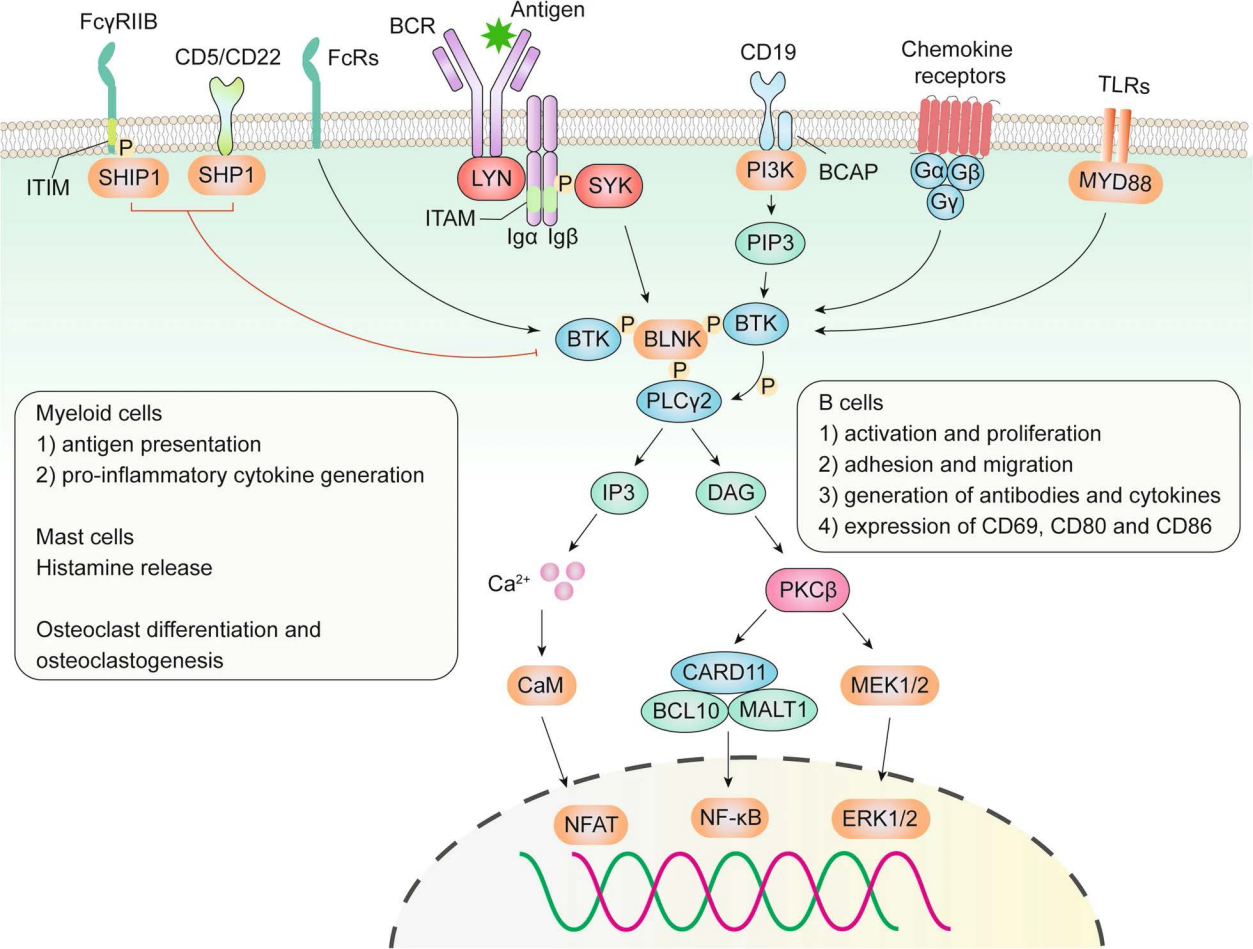
Role of BTK in the BCR, TLR, chemokine receptor, and Fc receptor (FcR) signaling pathways (8).

The BCR pathway is essential for the proliferation and survival of cancer cells in various B-cell malignancies, with BTK being a key component. BTK is a nonreceptor protein kinase belonging to the Tec family (**Figure 1**) (8). Originally identified as the faulty protein in X-linked agammaglobulinemia by Vetrie et al. in 1993, BTK is also referred to as agammaglobulinemia tyrosine kinase (9, 10). This kinase is expressed predominantly in cells of the hematopoietic lineage, including B cells, mast cells, and macrophages, but not in T cells, natural killer cells, or plasma cells (11). BTK is instrumental in multiple processes, such as B-cell lymphangiogenesis, as well as the development, maturation, and differentiation of immature B cells and their subsequent proliferation and survival (12, 13).

BTK is a key element within various B-cell receptors (BCRs), serving as a regulator of multiple intracellular signaling pathways mediated by cell surface molecules such as phosphoinositide 3-kinase (PI3K), mitogen-activated protein kinase (MAPK), and NF-κB. This regulation is crucial for the activation, proliferation, and differentiation of antibody-producing plasma cells (14, 15). As a result, the abnormal activation of BTK can lead to several B-cell malignancies, including various types of leukemias and lymphomas (16), as well as autoimmune disorders such as rheumatoid arthritis (RA) and multiple sclerosis (MS) (17).

**Figure 2:**
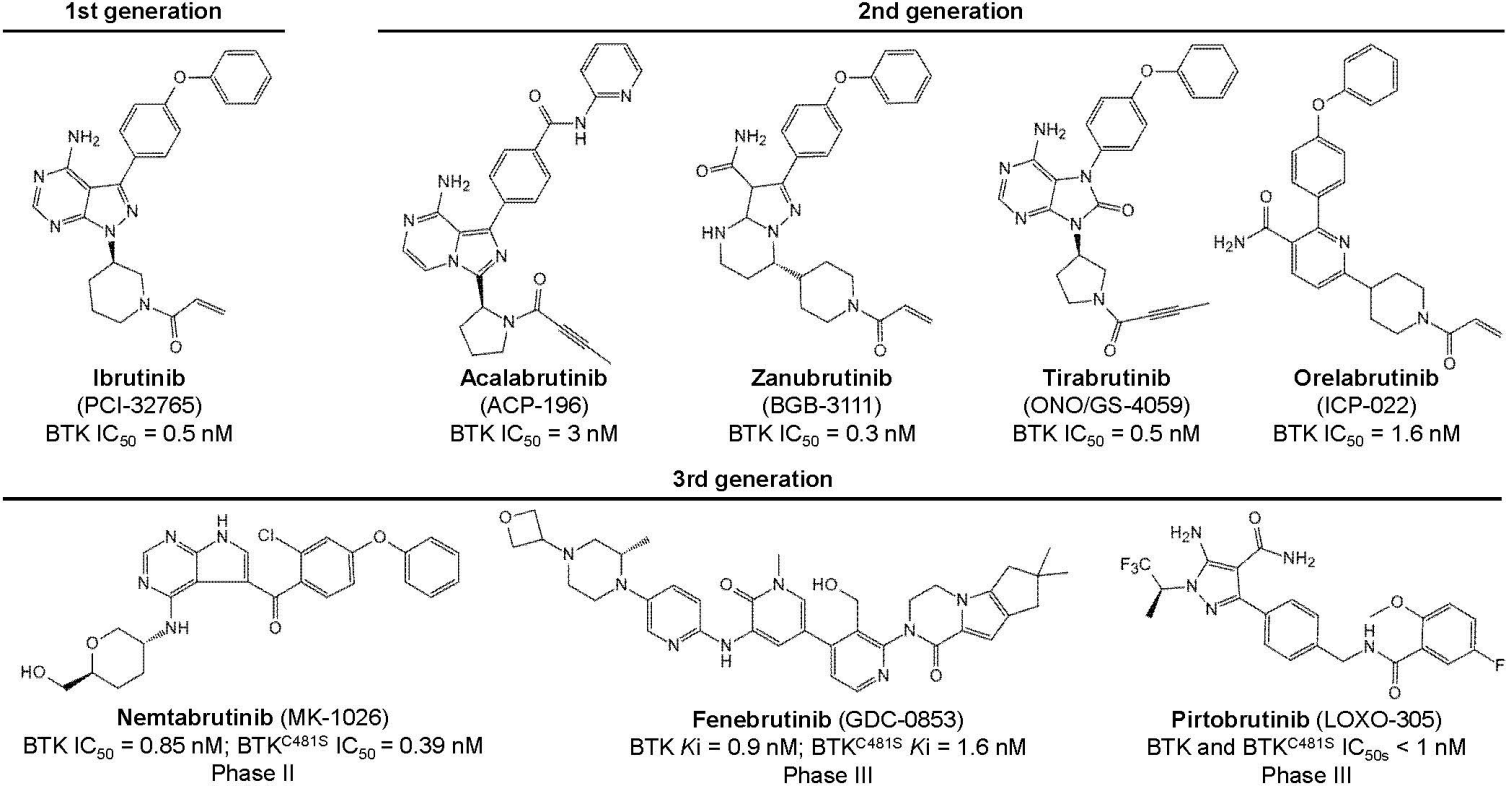
The structure and interactions of BTK (8).

BTK is composed of five distinct regions: the pleckstrin homology (PH) domain, the Tec homology (TH) domain, the SRC homology 2 (SH2) and 3 (SH3) domains, and a C-terminal catalytic domain (18, 19) (**Figure 2**) (8). The PH domain is crucial for mediating interactions with phospholipids and proteins. The TH domain, which contains two proline-rich regions (PRRs), plays a role in the autoregulation of BTK. The SH2 and SH3 domains are involved in binding to phosphorylated tyrosine residues and PRRs, respectively, with the SH3 domain also hosting a vital autophosphorylation site at tyrosine 223. The catalytic domain at the C-terminus, featuring the kinase activity center at tyrosine 551, is key for the initial activation of BTK (20). Additionally, within this catalytic domain, the residue Cys481 is a critical covalent binding site targeted by BTK inhibitors.

BTK activation occurs through the spleen tyrosine kinase (SYK), which itself is activated by the B-cell receptor (BCR) (20). Once activated, BTK phosphorylates residues Y753 and Y759 on phospholipase C beta 2 (PLCβ2), leading to the formation of inositol trisphosphate (IP3) and diacylglycerol (DAG). These secondary messengers then activate protein kinase C beta (PRKCB/PKCβ) rather than producing it. This results in elevated calcium levels, which then activate the MAPK/extracellular signal-regulated kinase (ERK) pathway, influencing the expression of genes related to cell proliferation, survival, and cytokine secretion. Concurrently, BTK can also activate the protein kinase B (AKT)/NF-κB signaling pathway (21, 22). Additionally, active BTK plays a role in mediating proinflammatory signals, including the production of inflammatory cytokines such as tumor necrosis factor (TNF)-α and interleukin 1b (IL1B), linking it to inflammatory responses (23).

### 2.2 Mechanisms of action of BTK inhibitors

As previously discussed, Cys481 within BTK is a nucleophilic site that forms a covalent bond with electrophilic inhibitors. Sequence analysis revealed that this cysteine is analogous to Cys773 in the epidermal growth factor receptor (EGFR) family, which has been targeted by several irreversible kinase inhibitors in clinical trials. This similarity inspired the development of a selective BTK inhibitor aimed at irreversibly deactivating this particular site. Through extensive research, scientists synthesized a compound that significantly reduced the phosphorylation of phospholipase C gamma 1 (PLCG1) associated with BTK and inhibited the LYN proto-oncogene Src family tyrosine kinase (LYN)- and spleen tyrosine kinase (SYK)-dependent phosphorylation at Tyr551 on BTK. This compound demonstrated high selectivity for BTK, with a marked preference over LYN or SYK, effectively and irreversibly inhibiting BTK-dependent pathways in the BCR signaling cascade. Consequently, these BTK inhibitors have attracted considerable attention (20).

BTK inhibitors are categorized into two types on the basis of their binding modes and mechanisms of action: irreversible and reversible. Irreversible inhibitors function through a Michael acceptor component that forms a covalent bond with the conserved Cys481 residue within the ATP-binding site, thereby permanently disabling the kinase. Conversely, some reversible inhibitors interact with specific pockets in the SH3 structural domain through noncovalent interactions such as hydrogen bonds or hydrophobic interactions, leading to an inactive kinase conformation (24). Some drugs such as pirtobrutinib blocks the ATP-binding site of BTK through non-covalent, non-C481-dependent binding, thereby overcoming acquired resistance to covalent BTK inhibitors (25). Most of the BTK inhibitors currently approved for use are irreversible (18). However, the clinical efficacy of these irreversible inhibitors, such as ibrutinib—the first of its kind to be marketed—has been compromised by the emergence of drug-resistant mutations. Notably, a mutation in which Cys481 is replaced by serine (C481S) diminishes the reactivity of BTK to ibrutinib and other covalent inhibitors, substantially reducing its effectiveness. For example, the inhibitory potency of ibrutinib is reduced sixfold against the C481S mutant (half-maximal inhibitory concentration = 4.6 nM). Other mutations at Cys481, including C481R, C481F, and C481Y, as well as mutations at the gatekeeper residue Thr474 (T474I, T474S, T474M), have been identified and pose similar challenges (26, 27). While ibrutinib can still bind noncovalently to the C481S mutant, this reversible interaction does not ensure sustained efficacy in patients harboring this mutation (28, 29).

In this context, noncovalent inhibitors that do not rely on interactions with Cys481 have been shown to effectively inhibit BTK mutants such as C481R, T474I, and T474M, representing promising therapeutic alternatives (26). Additionally, reversible inhibitors have demonstrated increased efficacy in the treatment of autoimmune diseases, including rheumatoid arthritis (RA), various forms of multiple sclerosis (MS), chronic graft-versus-host disease, and systemic lupus erythematosus (30, 31). Moreover, recent advancements have led to the development of protein hydrolysis-targeted chimeric molecules, introducing a novel strategy for reducing BTK activity (32).

### 2.3 Approved BTK inhibitors

**Figure 3:**
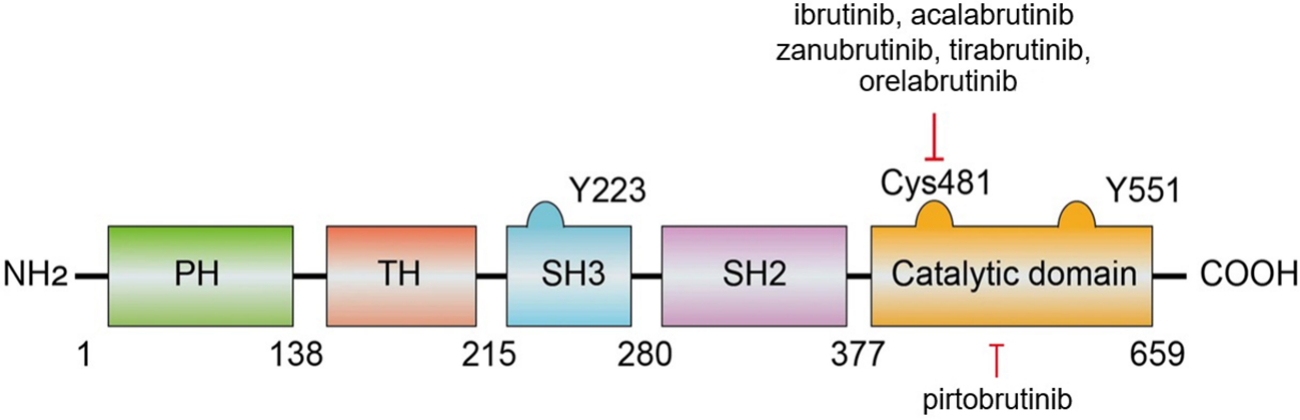
Chemical structures of the BTK inhibitors.

Six BTK inhibitors are currently approved and marketed worldwide: first-generation ibrutinib; second-generation acalabrutinib, zanubrutinib, tirabrutinib, and orelabrutinib; and third-generation molecules, including the newly marketed pirtobrutinib and the not-yet-marketed nemtabrutinib and fenebrutinib (**Figure 3**; **Table 1**). First- and second-generation BTK inhibitors irreversibly inhibit BTK activity by covalently binding to the Cys481 site in BTK. The third-generation BTK inhibitors are noncovalent inhibitors that do not depend on binding to Cys481 and still have a better inhibitory effect on BTK mutated at this site, with better efficacy and safety.

**Table 1:** Summary of approved BTK inhibitors.

Generation	BTK Inhibitor	Synonyms	Year of Approval	Type of Inhibitor	Indication
1^st^	Ibrutinib	PCI-32765	2013	Irreversible	MCL, CLL, SLL, WM, MZL, GVHD
2^nd^	Acalabrutinib	ACP-196	2017	Irreversible	CLL, SLL, MCL
	Zanubrutinib	BGB-3111	2019	Irreversible	NHL, CLL, MCL
	Tirabrutinib	ONO/GS-4059	2020	Irreversible	CNS lymphoma, WM, CLL
	Orelabrutinib	ICP-022	2020	Irreversible	MCL, CLL, SLL
3^rd^	Pirtobrutinib	LOXO-305	2023	Reversible	CLL/SLL, MCL

CLL, chronic lymphocytic leukemia; CNS, central nervous system; GVHD, graft-versus-host disease; MCL, mantle cell lymphoma; MZL, marginal zone lymphoma; SLL, small lymphocytic lymphoma; WM, Waldenström macroglobulinemia.

Ibrutinib, a pioneering and selective BTK inhibitor, was recognized as a breakthrough therapy by the U.S. Food and Drug Administration (FDA) in 2013, marking a significant milestone in medical treatment. It effectively inhibits tumor cell proliferation and impacts the adhesion and migration of malignant cells and the tumor microenvironment, which includes T and mesenchymal cells (33–37). In November 2013, the FDA initially approved ibrutinib for the treatment of mantle cell lymphoma (MCL). It was later approved to address various B-cell lymphomas, including chronic lymphocytic leukemia (CLL)/small lymphocytic lymphoma (SLL), Waldenström macroglobulinemia (WM), and marginal zone lymphoma (MZL). Despite its efficacy, resistance has developed in some patients who have relapsed following treatment with ibrutinib.

Research has indicated that certain baseline molecular and cytogenetic characteristics, such as del(17p)/tumor protein p53 (TP53) mutations and complex karyotypes (23 chromosomal abnormalities), increase the risk of disease progression in patients with primary chronic lymphocytic leukemia/small lymphocytic lymphoma (CLL/SLL) undergoing treatment with ibrutinib. Patients carrying the del(17p)/TP53 mutation are particularly prone to recurrence, which is often linked to BTK mutations (38, 39). Specifically, in relapsed/refractory (R/R) patients, the C481 residue of BTK is most commonly mutated to serine (C481S), which impedes the covalent attachment of ibrutinib to the structural domain of BTK (29, 40–42). Additionally, cells with the BTK T316A mutation exhibit resistance to ibrutinib, both at the cellular and molecular levels, comparable to the resistance observed with the C481S mutation (43). Moreover, missense mutations in PLCG2, a target of BTK, have emerged as another resistance mechanism. The activation of PLCG2 enables CLL cells to proliferate independently of BTK control, often cooccurring with BTK mutations to foster ibrutinib resistance (43). Another contributing factor to CLL resistance is del(8p), which results in the loss of TRAIL-R and significant epigenetic alterations (44). In response to these resistance issues and to mitigate side effects associated with ibrutinib, such as skin and dermatologic issues (45), bleeding, infections (46), headaches, and atrial fibrillation, newer generation BTK inhibitors, such as acalabrutinib and zanubrutinib, were developed and received FDA approval in 2017 and 2019, respectively (47). These advancements aim to offer more effective and tolerable treatment options for patients with CLL and related conditions.

The three approved BTK inhibitors—ibrutinib, zanubrutinib, and acalabrutinib—share similarities and differences in their action and usage. All three bind irreversibly and covalently to the Cys481 residue in the ATP-binding pocket of BTK. In terms of biochemical binding kinetics, ibrutinib is recognized as the most potent, followed by zanubrutinib and then acalabrutinib. Acalabrutinib stands out for having the highest selectivity and the lowest rate of off-target effects, followed by zanubrutinib, with ibrutinib having the least selectivity (48). Pharmacodynamic and pharmacokinetic variations among these inhibitors influence dosing, efficacy, and the profile of adverse effects observed in clinical practice. For example, acalabrutinib, with its shorter half-life and higher rate of BTK occupancy, is administered twice daily compared with ibrutinib’s once-daily regimen (95.3% vs. 87.6% BTK occupancy) (49). For zanubrutinib, studies have shown that a dosage of 160 mg twice daily achieves sustained complete suppression and more than 95% BTK occupancy in lymph nodes, making it more effective than a single daily dose of 320 mg. Thus, 160 mg twice daily has been recommended for further clinical studies (50). The interplay between rapid absorption and elimination in drug kinetics can lead to swift target inhibition while minimizing the risk of off-target effects and drug interactions. The selective properties and short half-life of acalabrutinib ensure thorough and continuous BTK inhibition, avoiding the toxicity associated with the inhibition of other kinases. Achieving full target engagement helps mitigate drug resistance arising from BTK mutations and lowers the incidence of Richter transformation (51).

Tirabrutinib is a highly selective and highly active BTK inhibitor developed in Japan for the treatment of recurrent or refractory primary central nervous system lymphoma (52). A phase 1/2 clinical study confirmed an overall response rate of 63.6% in patients with relapsed/refractory PCNSL treated with tirabrutinib. Common adverse reactions are rash, neutropenia, leukopenia, and lymphocytopenia (53). Orelabrutinib is a highly selective BTK inhibitor that is currently approved only for MCL and CLL in China (54). A phase I/II clinical study reported an overall response rate of 81.1% in patients with relapsed or refractory (r/r) mantle cell lymphoma (MCL). PMID: 37078706 Another phase 2 clinical study evaluating orelabrutinib (150 mg once daily) in patients with refractory or recurrent chronic lymphocytic leukemia (CLL)/small lymphocytic lymphoma (SLL). The total effective rate was 92.5% (74/80). Orelabrutinib is well tolerated, and the most common adverse reactions are decreases in leukocytes, hemoglobin and platelets and infection (55).

Recently, researchers have introduced pirtobrutinib, a reversible, noncovalent BTK inhibitor that is highly selective and effectively inhibits BTK phosphorylation, cellular proliferation, and tumor growth (56, 57). Unlike previous inhibitors, pirtobrutinib binds to BTK without Cys481, offering a potential solution to overcome the resistance observed with ibrutinib, acalabrutinib, and zanubrutinib. Nemtabrutinib is a multitarget inhibitor that can strongly inhibit the transmission of BCR signals. It can competitively occupy the ATP-binding pocket of BTK without interacting with C481, overcoming the resistance caused by this mutation. A phase I study demonstrated the initial efficacy of nemtabrutinib in patients with relapsed/refractory B-cell malignancies (58). The most common side effect of the drug is neutropenia. These new developments may represent significant advancements in the treatment of B-cell malignancies. Fenebrutinib is also a reversible BTK inhibitor that can both inhibit B-cell activation and reduce MS-induced inflammation. At present, related research in the field of multiple sclerosis is being carried out.

## 3 Clinical applications of BTK inhibitors for treating PCNSL

In the context of PCNSL, numerous studies have documented significant genomic alterations. Common genetic anomalies in PCNSL include single nucleotide and copy number variations, with the most frequently mutated genes being MYD88, CD79B, caspase recruitment domain family member 11 (*CARD11*), and TNF-α-induced protein 3 (*TNFAIP3*) (59). *MYD88* serves as a critical adaptor molecule in the Toll-like receptor (TLR) and interleukin 1 receptor type 1 (IL1R1) signaling pathways (60). Research by Ngo et al. indicated that activated B-cell-like diffuse large B-cell lymphoma (ABC-DLBCL) relies on MYD88, with the MYD88 L265P mutation being particularly prevalent in systemic DLBCL (61). This mutation is also commonly observed in PCNSL, making it the predominant subtype (62). *CD79B*, the second most frequently mutated gene in PCNSL, enhances B-cell receptor (BCR) signaling and NF-κB activation, thus providing survival signals to tumor cells (5, 63–65). Mutations in MYD88 L265P and CD79B are also utilized to classify ABC-DLBCL into distinct categories (66). Additionally, CARD11, which is a downstream component of the BCR pathway, has mutations that potentially activate NF-κB, contributing to the pathogenesis of PCNSL (67). Notably, mutations in CARD11 have been linked to resistance against the BTK inhibitor ibrutinib in several human B-cell malignancies (2, 29).

Recent research has highlighted that innovations targeting the BCR and TLR signaling pathways have been pivotal in advancing the treatment of PCNSL. These pathways offer various therapeutic targets. Upstream of the BCR signaling pathway, PI3K can be downregulated to suppress signaling. Some previously studied PI3K inhibitors have limited clinical use due to gastrointestinal toxicity. Amdizalisib, a novel highly selective PI3Kδ inhibitor, is currently undergoing clinical exploration for the treatment of hematologic malignancies (68). The drug has shown good safety and efficacy in patients with R/R lymphoma. Amdizalisib is not currently used in PCNSL, but owing to its good therapeutic effect and safety in other lymphomas, it may provide a new treatment option for patients with PCNSL. Another PI3K inhibitor, Linperlisib, a small-molecule inhibitor with high blood−brain barrier permeability, can effectively treat PCNSL and improve patient survival (69–71). Downstream, immunomodulatory drugs such as lenalidomide can inhibit interferon regulatory factor 4 (IRF4), thereby impacting NF-κB functionality. Proteasome inhibitors can also be used to prevent NF-κB from entering the nucleus, altering gene expression. However, a significant limitation of proteasome inhibitors is their inability to cross the blood−brain barrier, complicating their use in treating PCNSL (59). At the core of this pathway lies BTK, which is targeted effectively by BTK inhibitors.

### 3.1 First-generation BTK inhibitors

Ibrutinib is a first-generation BTK inhibitor. A phase I clinical trial by Gromes et al. (3) used ibrutinib monotherapy to treat 20 patients with R/R PCNSL, with an objective response rate (ORR) of 77%, including five patients with a complete response (CR), a PFS of 4.6 months, and an mOS of 15 months. In a further phase II clinical trial of single-agent ibrutinib in the treatment of R/R PCNSL and secondary CNS lymphoma (SCNSL), the ORR was 81%, the median PFS (mPFS) was four months, and the mOS was 19.5 months (72). In a phase II clinical trial (73) in which ibrutinib monotherapy was used in patients with R/R PCNSL or primary vitreoretinal lymphoma (PVSL), the ORR was 52%, the mPFS was 4.8 months, and the mOS was 19.2 months. These three studies demonstrated that ibrutinib monotherapy had a good clinical response in treating PCNSL.

Interestingly, the efficacy of BTK inhibitors in PCNSL is distinct from their performance in systemic lymphoma, where the single agent ibrutinib achieves only a 10% CR rate and a 15% PR rate (2). These findings underscore the limited impact of ibrutinib outside the CNS, in contrast with its more favorable outcomes in PCNSL, where despite a modest median PFS, the ORR is higher. This disparity may be attributed to the increased prevalence of BCR/TLR pathway alterations, such as mutations in MYD88, in PCNSL (73). Notably, even PCNSL patients without significant genomic alterations in the BCR signaling pathway respond to ibrutinib (73). However, the co-occurrence of CD79B and MYD88 mutations, while enhancing the sensitivity of systemic lymphomas to ibrutinib (2), does not seem to confer the same level of responsiveness in CNS disorders, potentially owing to a lesser reliance on the BCR signaling pathway in these conditions (3). These mutations are present in approximately 37% of PCNSL patients (64). Additionally, mutations in CARD11 and TNFAIP3, which operate downstream of BTK, have been identified as potential sources of resistance to ibrutinib in both systemic lymphoma (74) and PCNSL treated with ibrutinib monotherapy (3). This resistance is particularly relevant when considering the mechanisms of resistance that may arise when ibrutinib is used in combination with cytotoxic chemotherapy (65).

Since the efficacy of ibrutinib alone is often transient or incomplete, exploring its efficacy in combination with other antineoplastic drugs is meaningful. Gromes et al. (3) conducted three clinical trials. The first trial treated patients with PCNSL or SCNSL with ibrutinib, HD-MTX, and rituximab and reported an ORR of 89% (65). The second trial combined ibrutinib with copanisib to treat patients with R/R PCNSL and reported an ORR of 67% (75). The third trial combined 560–840 mg of ibrutinib with rituximab and lenalidomide to treat patients with R/R PCNSL or SCNSL and reported an mPFS of 3.03 months (76).

In 2017, Lionakis et al. (64) treated patients with R/R PCNSL via a regimen of ibrutinib with rituximab, liposomal adriamycin, temozolomide, etoposide, and dexamethasone (DA-TEDDi-R), with 86% achieving a CR and an overall efficacy rate of 94% and a PFS of 15.3 months. In 2020, Mark et al. (77) conducted a similar trial using ibrutinib in combination with temozolomide, etoposide, liposomal adriamycin, dexamethasone, and rituximab (TEDDi-R) to treat patients with R/R PCNSL, with one-year PFS and OS rates of 60.0% and 100%, respectively. In the same year, another study retrospectively analyzed 22 patients with R/R PCNSL treated with ibrutinib in combination with temozolomide and reported an ORR of 55% and a PFS of 11.7 months (78).

Despite high response rates to treatment, PFS with ibrutinib monotherapy is less than five months, suggesting the early emergence of resistance (79–81). Ibrutinib combination therapy extended PFS in pretreated patients to approximately nine months (65). Currently, a number of prospective studies are being conducted to combine ibrutinib with drugs such as lenalidomide, copanlisib, checkpoint inhibitors, and traditional chemotherapy. These results have not yet been published, but they provide new ideas and expectations for the application of BTK inhibitors in PCNSL. In conclusion, ibrutinib alone or in combination with chemotherapy has clear efficacy for the treatment of PCNSL. Combination chemotherapy is more efficacious than ibrutinib alone but also increases the risk of adverse effects, and the optimal combination approach still warrants further exploration.

### 3.2 Second-generation BTK inhibitors

Acalabrutinib, zanubrutinib, tirabrutinib, and orelabrutinib are second-generation BTK inhibitors, all of which are covalent. Zanubrutinib has greater target selectivity and fewer off-target effects than does ibrutinib. Zhang et al. (82) administered a zanubrutinib-containing regimen to four patients with newly diagnosed PCNSL and four patients with R/R PCNSL, and all patients with primary PCNSL and 75% of patients with R/R PCNSL achieved CR. Song et al. (83) combined rituximab, zanubrutinib, lenalidomide, and temozolomide with or without MTX (RLZT ± MTX) to treat PCNSL, achieving an ORR of 79.2%, making it a promising regimen for elderly patients who are intolerant of high-dose radiotherapy. A clinical study enrolled two groups of patients. One group included young patients in the RLZT+MTX group who received rituximab, lenalidomide, zanubrutinib, temozolomide, or high-dose methotrexate. The ORR of this group was 86.7% (CR rate 40%), and the other group of elderly patients was the RLZT group. The ORR of the RLZT group was 76.5% (CR rate 35.3%). This suggests that RLZT (without methotrexate) may be an option for older patients who cannot be treated with methotrexate (84). Another prospective study combined zanubrutinib with high-dose cytarabine to treat patients with R/R PCNSL, achieving an ORR of 75%, a median follow-up of 12 months, a PFS of 5.6 months, and an unmet mOS (85). All four studies used regimens combining zanubrutinib with other agents with favorable clinical responses. However, the results of zanubrutinib monotherapy have not been clarified, and an ongoing trial (NCT05117814) aimed to evaluate the efficacy and safety of zanubrutinib monotherapy in treating R/R PCNSL and SCNSL.

Four ongoing studies are examining the efficacy of acalabrutinib in treating PCNSL. One (NCT04688151) is a dose-escalation trial combining acalabrutinib with durvalumab to treat R/R PCNSL or SCNSL. The second is a trial combining acalabrutinib with rituximab and durvalumab to treat R/R PCNSL. The remaining two trials (NCT04906902 and NCT04548648) are designed to evaluate the safety and efficacy of acalabrutinib monotherapy for treating R/R PCNSL.

Tirabrutinib was the first BTK approved globally to treat R/R PCNSL. One study reported that elderly patients with PCNSL were switched to maintenance therapy with tirabrutinib after being unable to continue HD-MTX treatment, with their tumors almost disappearing and their cognitive function improving after two months (86). A phase I/II clinical trial in which single-agent tirabrutinib was used to treat patients with R/R PCNSL achieved an ORR of 64% and an mPFS of 2.9 months (53). Kawasaki et al. (87) conducted a prospective study evaluating tirabrutinib for R/R PCNSL, achieving an ORR of 63.0%. While the above studies suggest that tirabrutinib has a favorable clinical response in treating R/R PCNSL alone, the efficacy of combining it with other chemotherapeutic agents remains unknown. An ongoing trial (NCT04947319) is combining tirabrutinib with two different HD-MTX regimens (MTX, temozolomide, and rituximab or MTX, rituximab, procarbazine, and vincristine) to treat PCNSL and evaluate the safety and efficacy of tirabrutinib in combination with other chemotherapeutic agents.

Orelabrutinib has higher cerebrospinal fluid concentrations than ibrutinib does. A retrospective study evaluated the efficacy of orelabrutinib monotherapy in treating PCNSL, achieving a six-month OS rate of 100% (88). A clinical study enrolled patients with PCNSL who were treated with rituximab plus high-dose methotrexate and orelabrutinib. On day 15 of orelibrutinib treatment, the patients underwent a lumbar puncture, and the concentration of orelabrutinib in the cerebrospinal fluid was measured. One study confirmed that the cerebrospinal fluid concentration of orelabrutinib was much greater than that of drugs such as ibrutinib (89). Several recent clinical studies have examined the combination of orelabrutinib with other chemotherapy drugs to treat PCNSL. Yang et al. (90) treated 15 R/R PCNSL patients with orelabrutinib combined with rituximab, HD-MTX, temozolomide, and lenalidomide, achieving an ORR of 86.7% and a CR rate of 73.3%. Another phase II clinical trial achieved an ORR of 100% when orelabrutinib was combined with HD-MTX and rituximab to treat patients with newly diagnosed PCNSL (91). A retrospective study by Zeng et al. (92) evaluated the efficacy and safety of a regimen of tiotropium, orelabrutinib, and MTX with or without rituximab (TOM ± R) in treating patients with PCNSL, achieving an ORR of 92.3%, a CR rate of 53.9%, and a six-month PFS of 63.6%. Zhao et al. (93) administered orelabrutinib, rituximab, and HD-MTX to 34 patients newly diagnosed with PCNSL, achieving an ORR of 94.4% and a CR rate of 88.9%. A phase I/II clinical trial by Zhang et al. (94) evaluated the safety and efficacy of combining orelabrutinib, an anti-programmed cell death 1 (PDCD1/PD-1) antibody, and formostatin to treat patients with newly diagnosed PCNSL, achieving an ORR of 88.9% in a phase I study, with a phase II study still ongoing.

The five BTK inhibitors currently on the market are all covalent inhibitors that are prone to drug-resistant mutations and adverse reactions caused by off-target effects. The most commonly reported adverse reactions to ibrutinib include diarrhea, bleeding, atrial fibrillation, and infection, which may lead to treatment discontinuation in severe cases (**Table 2**). One study reported that 56% of patients treated with the single agent ibrutinib and 52% of patients treated with combination therapy developed infections (46). Among more than 500 patients who received ibrutinib for malignancy between 2009 and mid-2016, more than 75% developed new or worsening hypertension within a median of 30 months (46). Second-generation covalent BTK inhibitors mostly have higher target selectivity and less off-target toxicity, but they can still cause adverse reactions, such as headache, diarrhea, and infection. The risk of adverse reactions increases when some chemotherapy drugs are used together.

**Table 2:** Summary of efficacy and common adverse reactions of 5 BTK inhibitors.

Type	Treatment	Patient (*n*)	Efficacy			Adverse reactions	Reference
			ORR (%)	mPFS (m)	mOS (m)		
PCNSL	Ibrutinib	13	77.0	4.6	15.0	Lymphopenia, neutropenia, hyperglycemia, thrombocytopenia	(8)
	Ibrutinib + HD-MTX + Rituximab	9	89.0	9.2	not reached	Lymphopenia, lung infection, thrombocytopenia, hyperglycemia	(5)
	RLZT±MTX	24	79.2	not reached	not reached	Thrombocytopenia, leukopenia	(82)
	Orelabrutinib	23	100	9.80	not reached	Thrombocytopenia, leukopenia, rash	(53)
	Orelabrutinib + HD-MTX + Rituximab	10	100	not reached	not reached	Neutropenia, lymphopenia	(88)
	TOM±R	13	92.3	not reached	not reached	Leukopenia, thrombocytopenia, fever, pneumonia	(89)
	Orelabrutinib +Rituximab + HD-MTX	34	94.4	not reached	not reached	Leukopenia	(90)
	Orelabrutinib + anti-PD-1 antibody + Formustine	8	88.9	not reached	not reached	interstitial pneumonia	(91)
R/R PCNSL	Ibrutinib	29	81.0	4.0	19.5	Lymphopenia, neutropenia, elevated ALT, hyperglycemia, thrombocytopenia	(70)
	DA-TEDDi-R	18	86.0	15.3	not reached	Neutropenia, thrombocytopenia, pulmonary infection, aspergillosis	(65)
	TEDDi-R	13	75.0	not reached	not reached	Neutropenia, thrombocytopenia	(75)
	Ibrutinib + Temozolomide	22	55.0	11.7	8.9	infection	(76)
	Zanubrutinib + Cytarabine	12	75.0	5.6	not reached	Thrombocytopenia	(83)
	Tirabrutinib	44	64.0	2.90	not reached	Neutropenia, lymphopenia, hyperglycemia, elevated ALT	(85)
	Orelabrutinib + Rituximab + HD-MTX + Temozolomide + Lenalidomide	15	86.7	not reached	not reached	Elevated transaminase, decreased leukocytes	(87)

PCNSL, primary central nervous system lymphoma; R/R, relapsed/refractory; HD-MTX, high-dose methotrexate; TOM±R, tiotropium, orelabrutinib, and MTX with or without rituximab; DA-TEDDi-R, dose-adjusted temozolomide, etoposide, liposomal adriamycin, dexamethasone, ibrutinib and rituximab; ALT, alanine aminotransferase.

There are a number of other BTK inhibitors that have been reported. TL-895 is a very potent and highly selective BTK inhibitor (95). Another BTK inhibitor, M7583, has been shown to be effective in treating human B-cell malignancies (96). In addition, a Phase I clinical study demonstrated that the BTK inhibitor DTRMWXHS-12 in combination with everolimus and pomadomide was tolerated and clinically active (97). These drugs are expected to be further studied in the future. The compound RSH-7 strongly inhibits BTK and FLT3 signaling pathways by up-regulating proapoptotic protein and down-regulating Bcl-2 levels, and effectively inhibits the proliferation of various hematologic malignant tumor cells. RSH-7 may be a promising compound for the treatment of hematological malignancies (98). Preclinical studies have confirmed that BGB-8035 is highly selective and a promising preclinical candidate compound for the treatment of autoimmune diseases and B-cell lymphoma (99). JNJ-64264681 is a covalent, irreversible BTK inhibitor that has shown good oral efficacy in both cancer and autoimmune models and has entered human clinical studies (100). Studies have confirmed that AS-1763 has a significant effect on the *in vivo* xenogenic tumor model, and has entered the phase I clinical study (101). BTK inhibitors such as NX-2127, HPCL-760, and CG-806 can destroy B-cell receptors and inhibit B-cell pathways, which is expected to be further explored in the future (102, 103). Currently ongoing clinical trails of BTK inhibitors are shown in **Table 3**.

**Table 3:** Summary of ongoing clinical trials of BTK inhibitors.

Experimental drug	Development stage	Condition	Status	NCT number
Copanlisib + Ibrutinib	Phase IB/II	R/R PCNSL	Active, not recruiting	NCT03581942
Orelabrutinib + PD-1+ fotemustine	Phase I/II	PCNSL	Unknown status	NCT04831658
Ibrutinib+R-VMP	Phase I/II	PCNSL, SCNSL	Recruiting	NCT02315326
Ibrutinib + Rituximab + Lenalidomide	Phase I	R/R PCNSL, R/R SCNSL	Active, not recruiting	NCT03703167
Tirabrutinib	Phase II	PCNSL	Recruiting	NCT04947319
Ibrutinib+ CA-4948	Phase I/II	R/R PCNSL	Recruiting	NCT03328078

PD-1, programmed death receptor 1; R-VMP rituximab, methotrexate, vincristine, and procarbazine; R/R, relapsed/refractory; PCNSL, primary central nervous system lymphoma; SCNSL, secondary Central Nervous System Lymphoma.

Another concern is the issue of resistance. First- and second-generation BTK inhibitors inhibit BTK kinase activity by binding to its ATP-binding site and then covalently modifying Cys481. Most often, mutations convert this active cysteine to serine (C481S) and, less frequently, to phenylalanine (C481F), tyrosine (C481Y), or arginine (C481R), and the L528W mutation inactivates BTK. The growth and survival of DLBCL cells with kinase-naïve BTK are dependent on Toll-like receptor 9 (TLR9), leading to resistance to BTK inhibitors (104). While second-generation BTK inhibitors have shown better BTK selectivity and less off-target toxicity, they cannot reverse the resistance of tumor cells to ibrutinib. In conclusion, the role of BTK inhibitors in PCNSL is still in the clinical trial stage. While some clinical data are promising, they remain limited due to small sample sizes and a lack of blinding, randomized control, and comparison. Most phase III clinical trials on BTK inhibitors in PCNSL are ongoing, and we expect that more evidence will be available to confirm the efficacy, safety, and resistance of BTK inhibitors in PCNSL, with the goal of providing a basis for individualized treatment of patients with PCNSL in the real world.

## 4 Conclusion

Since PCNSL have similar biological properties and therapeutic responses, BTK inhibitors provide a new option for their treatment. Compared with first-generation BTK inhibitors, second-generation BTK inhibitors have significantly fewer off-target effects and fewer adverse effects, further improving patient prognosis. However, drug-resistant mutations in tumor cells remain a challenge. Pirtobrutinib, the only approved third-generation noncovalent BTK inhibitor, is used to treat R/R lymphomas after at least two lines of prior systemic therapy, and the effectiveness and efficacy of noncovalent BTK inhibitors in treating PCNSL remain unknown. Further clinical studies examining the efficacy of BTK inhibitors in treating PCNSL are needed. The therapeutic efficacy of BTK inhibitors as monotherapies is usually short-lived and incomplete, and the combination of BTK inhibitors with other treatments may increase their adverse effects while further improving patient prognosis. However, combining BTK inhibitors may solve the problem of drug resistance, and exploring the development of BTK inhibitor-based combination therapy to further improve the efficacy and safety of treating PCNSL is worthwhile.

## 5 Future directions

Novel BTK inhibitors, immunomodulators, anti-PD-1 drugs, CAR-T cells, etc., have provided new options for the treatment of PCNSL patients. However, more clinical studies on long-term efficacy and survival are needed. Promising therapeutic strategies should be actively developed, and more drug combinations should be explored to provide further reference for clinical practice. Increased expression or activity of BTK has been associated with increased blood-brain barrier permeability in several studies, and orelabrutinib has shown potential in the treatment of central system lymphoma. In the development of a new generation of BTK inhibitors, improving the blood-brain barrier permeability of BTK inhibitors is an important direction.
